# Sociodemographic inequalities in cigarette, smokeless tobacco, waterpipe tobacco, and electronic cigarette use among adolescents aged 12–16 years in 114 countries: A cross-sectional analysis

**DOI:** 10.18332/tid/191824

**Published:** 2024-09-02

**Authors:** Mohammed Jawad, Wei Li, Filippos T. Filippidis

**Affiliations:** 1 Department of Primary Care and Public Health, School of Public Health, Imperial College London, London, United Kingdom

**Keywords:** tobacco, cigarettes, smoking, adolescents, inequalities

## Abstract

**INTRODUCTION:**

The majority of users of tobacco and nicotine products start using them in adolescence. In order to keep equity considerations at the forefront of tobacco control, it is crucial to assess whether inequalities in prevalence of tobacco and nicotine use exist among adolescents globally.

**METHODS:**

We conducted a secondary analysis of Global Youth Tobacco Survey (GYTS) data from 538644 school-based adolescents (79.3% aged 13–15 years) in 114 countries (2013–2019). Data were collected on current (past 30-day) use of cigarettes, smokeless tobacco, waterpipe tobacco and electronic cigarettes. We used weighted Poisson regression models adjusted for sex, pocket money, and age to assess differences in prevalence of current use between boys and girls, and between students with high versus low pocket money.

**RESULTS:**

Although there was substantial regional variation, in most countries boys were statistically significantly more likely to report current use of all assessed products (ranging from 50.0% of countries for waterpipe tobacco to 73.3% of countries for electronic cigarettes). Inequalities by sex were less pronounced in Europe compared to other regions. Inequalities by pocket money were less consistent; students with more pocket money were more likely to report current use of cigarettes (vs those with less pocket money) in 61.8% of the countries, but more likely to report current use of smokeless tobacco in only 18.3% of countries.

**CONCLUSIONS:**

Globally, boys and adolescents with more pocket money are generally more likely to use a range of tobacco and nicotine products. However, these patterns are not universal and local variations should be taken into consideration to design effective and equitable tobacco control policies.

## INTRODUCTION

Tobacco use is estimated to kill approximately 7.7 million people each year, the majority in low- and middle-income countries^1^. Among the 1.14 billion smokers in the world, the vast majority started using tobacco in adolescence or early adulthood^1,^ ^2^, which explains the intense interest of both the public health community and the tobacco industry in youth. Tobacco use is associated with substantial health risks for all users, but it has been shown to be particularly harmful for adolescents. Smokers who start smoking at an early age are more heavily depended on nicotine, less likely to quit, and face increased mortality compared to smokers who started smoking later^3,4^. In addition, adolescence is a crucial age for body and brain development which nicotine and tobacco use negatively affect^5^.

The use of tobacco and other nicotine products is a complex behavior with many contributing factors. Among adolescents, the interaction between sociodemographic and environmental factors can heavily influence the likelihood of experimenting with and regularly using cigarettes or other nicotine products. These factors include gender^6^, socioeconomic status^7^, peer and parental influence^2^, ethnicity^8^, stress and mental health^9^, affordability of tobacco products^10^, exposure to direct and indirect tobacco marketing^11,12^ and implementation of tobacco control policies^13,14^.

These factors vary considerably both within and between countries, which has led to substantial inequalities in tobacco and nicotine use among youth and adults. Although differences between countries are well-established and relatively easy to monitor due to a wealth of nationally representative surveys^1^, studies on inequalities by sex and – especially – socioeconomic status (SES), often focus on a single country or region and the majority are conducted in adult populations. However, tobacco use prevalence in adult populations encompasses multiple generations and is a function of both initiation and cessation of tobacco use over decades. As such, it might not reflect current circumstances and may not be informative of differences in tobacco and nicotine use among adolescents. This has been particularly true in recent years with the introduction of novel tobacco and nicotine products, such as the electronic cigarettes. Electronic cigarettes are popular among young people who have been increasingly experimenting with them in many parts of the world^15^. We have also observed a resurgence of more traditional tobacco products in certain countries among youth, including waterpipe and smokeless tobacco^14,16^.

The aim of this study was to assess differences in the prevalence of current use of cigarettes, waterpipe tobacco, smokeless tobacco and electronic cigarettes by sex and by SES, among adolescents globally.

## METHODS

### Sample and data

We conducted a secondary analysis of the Global Youth Tobacco Surveys (GYTS). These are cross-sectional, nationally representative, self-administered, school-based surveys completed by adolescents typically aged 13–15 years worldwide, although younger and older students are occasionally included in the survey samples. Standardized questionnaires and sampling methodologies are employed that enable comparisons between countries and over time. GYTS participants are selected in a two-stage cluster design, where schools are selected with a probability proportional to their sizes and classes are selected with equal probability. Individual level analysis weights, which include sample selection and post-stratification factors, were provided for each country. More details on the GYTS methodology can be found elsewhere^17^. We included only the most recent survey from each country for which data were available at the time of data extraction (June 2021). We excluded surveys that were conducted at the sub-national level or in sub-populations, those conducted before the year 2013, and those that did not ask questions about pocket money. This study was exempt from ethics approval as the data used are publicly available and anonymized.

### Measures

The four primary outcome measures were current (past 30-day) use of cigarettes, smokeless tobacco, waterpipe tobacco, and electronic cigarettes. All GYTS datasets include current cigarette use, however the inclusion of other products is at the discretion of individual countries. Sociodemographic variables of interest were sex (male/female) and pocket money. Pocket money was assessed with the question: During an average week, how much money do you have that you can spend on yourself, however you want?’. The response options were: ‘I usually don’t have any spending money’, followed in most cases by six monetary ranges that differed in each country depending on socioeconomic contexts and local currency. As pocket money responses were not comparable across countries, we standardized each country’s responses into three categories: low socioeconomic status (approximately the quartile with the least amount of pocket money), high socioeconomic status (the quartile with the most amount of pocket money), and moderate socioeconomic status (the two middle quartiles).

### Statistical analysis

Given that all four outcomes followed a Poisson distribution, we assessed sex and socioeconomic inequalities for each country and outcome using survey-weighted Poisson regression models. Each model included age, sex and SES as independent variables. These models produce a prevalence ratio (PR) with females as the reference group for sex and low SES as the reference group for pocket money. For pocket money we present only the high versus low SES PR for simplicity. To prevent biased estimates, we excluded countries where <0.5% prevalence was documented in sex and pocket money cross-tabulations; for cigarette use, these included Bangladesh, Cambodia, Sri Lanka, and Tajikistan; for smokeless tobacco use, these included Argentina, Belarus, Kazakhstan, Niue, and Tokelau; for waterpipe use, this included Togo. No country was excluded for electronic cigarette use.

All PRs are presented as coefficients with 95% confidence intervals (95% CIs) in forest plots stratified by World Health Organization regions: African (AFR), Americas (AMR), South-East Asian (SEAR), European (EUR), Eastern Mediterranean (EMR), and Western Pacific (WPR). All statistical analyses were conducted on Stata (version 15.1).

## RESULTS

### Sample characteristics

Our final sample included 538644 adolescents from 114 countries (Supplementary file Table S1). An unweighted total of 10.3% of participants from 110 countries were current cigarette users. The respective numbers – in a lower number of countries – were 4.9% for current smokeless tobacco users (104 countries), 10.7% for current waterpipe users (50 countries), and 9.5% for current electronic cigarette users (60 countries). The sample size and survey year for each country are shown in Supplementary file Table S1. Most adolescents were aged 13–15 years (79.3%) and 51.3% were female. Just under a quarter (23.8%) were classified as high SES, 20.7% as low SES, and 55.5% as moderate SES. Table 1 and Supplementary file Table S2 show a summary of results by country.

**Table 1:** Summary of Poisson regression results on inequalities of cigarette, smokeless tobacco, waterpipe and e-cigarette use in 114 countries

*Regions*	*Inequalities by sex*			*Inequalities by SES*		
	*Girls report higher prevalence n (%)*	*Boys report higher prevalence n (%)*	*No statistically significant difference n (%)*	*Low SES report higher prevalence n (%)*	*High SES report higher prevalence n (%)*	*No statistically significant difference n (%)*
**Cigarettes**						
AFR (n=19)	0 (0.0)	14 (73.7)	5 (26.3)	1 (5.3)	11 (57.9)	7 (36.8)
AMR (n=24)	0 (0.0)	12 (50.0)	12 (50.0)	0 (0.0)	9 (37.5)	15 (62.5)
EMR (n=16)	0 (0)	16 (100)	0 (0)	0 (0)	7 (43.8)	9 (56.3)
EUR (n=27)	4 (14.8)	10 (37.0)	13 (48.1)	0 (0.0)	24 (88.9)	3 (11.1)
SEAR (n=6)	0 (0)	6 (100)	0 (0)	0 (0)	4 (66.7)	2 (33.3)
WPR (n=18)	0 (0.0)	16 (88.9)	2 (11.1)	0 (0.0)	13 (72.2)	5 (27.8)
Total (n=110)	4 (3.6)	74 (67.3)	32 (29.1)	1 (1.0)	68 (61.8)	41 (37.3)
**Smokeless tobacco**						
AFR (n=17)	0 (0.0)	6 (35.3)	11 (64.7)	2 (11.8)	5 (29.4)	10 (58.8)
AMR (n=23)	0 (0.0)	7 (30.4)	16 (69.6)	2 (8.7)	0 (0.0)	21 (91.3)
EMR (n=16)	0 (0.0)	9 (56.3)	7 (43.8)	3 (18.8)	2 (12.5)	11 (68.8)
EUR (n=23)	0 (0.0)	16 (69.6)	7 (30.4)	2 (8.7)	5 (21.7)	16 (69.6)
SEAR (n=8)	0 (0.0)	6 (75.0)	2 (25.0)	1 (12.5)	4 (50.0)	3 (37.5)
WPR (n=17)	0 (0.0)	10 (58.8)	7 (41.2)	1 (5.9)	3 (17.6)	13 (76.5)
Total (n=104)	0 (0.0)	54 (51.9)	50 (48.1)	11 (10.6)	19 (18.3)	74 (71.2)
**Waterpipe tobacco**						
AFR (n=9)	0 (0.0)	2 (22.2)	7 (77.8)	1 (11.1)	3 (33.3)	5 (55.6)
AMR (n=12)	0 (0.0)	4 (33.3)	8 (66.7)	0 (0.0)	3 (25.0)	9 (75.0)
EMR (n=14)	1 (7.1)	11 (78.6)	2 (14.3)	0 (0.0)	5 (35.7)	9 (64.3)
EUR (n=11)	0 (0.0)	5 (45.5)	6 (54.5)	0 (0.0)	10 (90.9)	1 (9.1)
SEAR (n=2)	0 (0)	2 (100)	0 (0)	0 (0)	2 (100)	0 (0)
WPR (n=2)	0 (0.0)	1 (50.0)	1 (50.0)	0 (0.0)	1 (50.0)	1 (50.0)
Total (n=50)	1 (2.0)	25 (50.0)	24 (48.0)	1 (2.0)	24 (48.0)	25 (50.0)
**Electronic cigarettes**						
AFR (n=6)	0 (0.0)	2 (33.3)	4 (66.7)	1 (16.7)	3 (50.0)	2 (33.3)
AMR (n=19)	0 (0.0)	12 (63.2)	7 (36.8)	0 (0.0)	6 (31.6)	13 (68.4)
EMR (n=5)	0 (0)	5 (100)	0 (0)	0 (0)	2 (40.0)	3 (60.0)
EUR (n=18)	0 (0.0)	15 (83.3)	3 (16.7)	0 (0.0)	16 (88.9)	2 (11.1)
SEAR (n=1)	0 (0)	1 (100)	0 (0)	0 (0)	0 (0)	1 (100)
WPR (n=11)	0 (0.0)	9 (81.8)	2 (18.2)	0 (0.0)	3 (27.3)	8 (72.7)
Total (n=60)	0 (0.0)	44 (73.3)	16 (26.7)	1 (1.7)	30 (50.0)	29 (48.3)

Results shown for inequalities by sex are from Poisson regression models adjusted for age and socioeconomic status (SES). Results shown for inequalities by SES are from Poisson regression models adjusted for age and sex. AFR: African Region. AMR: Americas Region. SEAR: South-East Asian Region. EUR: European Region. EMR: Eastern Mediterranean Region. WPR: Western Pacific Region. SES: socioeconomic status.

### Inequalities by sex

Inequalities by sex in 110 countries that reported current cigarette use are shown in Supplementary file Figure S1, where PRs ranged from 0.61 in Uruguay (95% CI: 0.28–1.33) to 17.69 in Indonesia (95% CI: 12.66–24.64). About two-thirds of countries (n=74) reported significantly higher prevalence of cigarette use in males than females in our age- and SES-adjusted models, whereas only four countries, all in the EUR (Bulgaria, Italy, Slovakia, and Slovenia), reported higher use in females (Table 1). All 16 countries in the EMR, all six countries in the SEAR, and 16 of 18 countries in the WPR reported significantly higher prevalence in males.

Inequalities by sex in current smokeless tobacco use were assessed in 104 countries (Supplementary file Figure S2), where PRs ranged from 0.69 in Tajikistan (95% CI: 0.40–1.19) to 6.45 in Myanmar (95% CI: 3.99–10.44). About half of countries (n=54) reported significantly higher prevalence in males, and no countries reported higher prevalence in females (Table 1). The EUR (n=16; 69.6%) and SEAR (n=6; 75.0%) had the highest percentage of countries where males use smokeless tobacco more than females.

Current waterpipe tobacco use was assessed in 50 countries (Supplementary file Figure S3), where PRs ranged from 0.74 in Djibouti (95% CI: 0.56–0.98) to 5.10 in Tunisia (95% CI: 3.18–8.17). Half of the countries (n=25) reported significantly higher prevalence in males, and only one country (Djibouti) reported higher prevalence in females (Table 1). The EMR (n=11; 78.6%) and SEAR (n=2; 100.0%) had the highest percentage of countries where male adolescents use waterpipe tobacco more than females.

Inequalities by sex in 60 countries that reported current electronic cigarette use are shown in Supplementary file Figure S4. PRs for males versus females ranged from 0.88 in Ghana (95% CI: 0.54–1.44) to 6.09 in Mongolia (95% CI: 3.04–12.21). Forty-four countries reported significantly higher prevalence in males, and no countries reported higher prevalence in females (Table 1). In EMR (n=5) and SEAR (n=1) males reported higher prevalence of current electronic cigarette use than females in all participating countries.

### Inequalities by socioeconomic status

Inequalities by SES for current cigarette use are shown in Supplementary file Figure S5, where PRs ranged from 0.48 in Ghana (95% CI: 0.18–1.32) to 7.24 in Tanzania (95% CI: 1.50–34.83). Sixty-eight of the 110 countries reported significantly higher prevalence of current cigarette use in students with high SES, whereas only one country (Mauritania), reported higher prevalence in students with low SES (Table 1). The EUR (n=24; 88.9%), and WPR (n=13; 72.2%) were the two regions with the highest proportion of countries where cigarette use was higher in students with high SES.

Supplementary file Figure S6 presents inequalities by SES for current smokeless tobacco use. PRs ranged from 0.11 in Uruguay (95% CI: 0.04–0.32) to 10.07 in Sri Lanka (95% CI: 4.26–23.78). Only 19 countries (18.3%) reported significantly higher prevalence in students with high SES, compared to 11 countries (10.6%) that reported higher prevalence in low SES groups (Table 1). Most countries (71.2%) reported no difference between SES groups. Reports of higher prevalence of smokeless tobacco in students with high compared to low SES were more frequent in AFR (n=5; 29.4%) and SEAR (n=4; 50.0%).

Regarding current waterpipe tobacco use (Supplementary file Figure S7), PRs ranged from 0.74 in Djibouti (95% CI: 0.56–0.98) to 5.10 in Tunisia (95% CI: 3.18–8.17). Under half of countries (n=24; 48.0%) reported significantly higher prevalence in students with high SES and half (n=25) reported no difference in prevalence between SES groups (Table 1). In all, 90.9% of EUR countries (n=10) and both SEAR countries included in the analysis reported higher prevalence of current waterpipe tobacco use in students with high SES.

Prevalence ratios of current electronic cigarette use by SES are shown in Supplementary file Figure S8. Among 60 assessed countries, PRs ranged from 0.31 in Ghana (95% CI: 0.14–0.69) to 7.85 in San Marino (95% CI: 2.03–30.42). Half of countries (n=30) reported significantly higher use in students with high SES, and just under half (n=29) reported no difference in use between SES groups (Table 1). The EUR (n=16; 88.9%) had the highest percentage of countries where prevalence of electronic cigarette use was higher in students with high SES.

## DISCUSSION

This analysis of over half a million adolescents from 114 countries showed that inequalities by sex and SES vary across products and between countries and regions. A predominant pattern was increased prevalence of tobacco and nicotine use in boys (ranging from 50.0% of countries for waterpipe tobacco to 73.3% of countries for electronic cigarettes) although a substantial percentage of countries reported no difference by sex (ranging from 26.7% for electronic cigarettes to 48.1% for smokeless tobacco). Only in five countries did females use tobacco more than males; four countries from the EUR reporting cigarette use and one country from the EMR reporting waterpipe tobacco use. Patterns by SES were less consistent; students with high SES had increased use compared to those with low SES in 18.3% of countries reporting smokeless tobacco use, 48.0% reporting waterpipe tobacco, 50.0% reporting electronic cigarette use, and 61.8% reporting cigarette use. Among all the comparisons we conducted, only fourteen showed higher prevalence in low SES groups, eleven of which were in cases of smokeless tobacco.

Sex inequalities in tobacco use are not surprising. In most countries and for most of the past century, the prevalence of smoking among men has been higher than women^1^. Although this gap has been decreasing in many high-income settings, our analysis showed that it still persists among adolescents. As expected, the differences are highest in regions such as EMR and SEAR where, traditionally, social acceptability of tobacco use by women has been low. Even within the same geographical region though, cultural and market factors may create stark differences. For instance, there seems to be a clear divide between the European Union (EU) and the rest of the countries of the EUR; there are very few EU countries in which boys reported higher prevalence of any tobacco product and there are indeed some where girls were more likely to smoke cigarettes than boys. We had no consistently collected data on the frequency of use, which might still differ by sex in the EU^18^; however, these findings highlight cultural differences in societal perceptions of tobacco use across the globe which influence adolescents’ behaviours^19^.

Interestingly, these patterns, which are well known for cigarette smoking, seem to be even stronger with regard to electronic cigarettes. Although novel tobacco products are being marketed by the tobacco industry as ‘less harmful’ and more ‘fashionable’ than traditional cigarettes^20^, they seem to appeal more to adolescent boys than girls. Considering that this is also true for waterpipe and smokeless tobacco^14-16^, it is challenging to disentangle the role of culture, social perceptions and the tobacco industry’s marketing efforts in creating these inequalities. Nevertheless, our analysis showed that these differences are not universal and may vary in magnitude; therefore, it is crucial to explore the local context when designing tobacco control policies and interventions to prevent tobacco and nicotine use among youth.

The findings on the association between SES and tobacco and nicotine use were more variable across countries and regions and, arguably, less consistent with existing literature. The majority of studies on SES and smoking have shown that people in disadvantaged groups are more likely to be smokers compared to those with higher education and/or income^7,21^. These studies are mostly done in high-income settings; findings from low- and middle-income countries are less consistent^7^, although they frequently reveal similar patterns^21,22^. However, these inequalities by SES might not emerge until later in life; studies focusing on children and adolescents do not necessarily show such differences^21^. Based on our definition of current use (i.e. any use in the past 30 days) and the nature of substance use behaviors in adolescence, we may have captured students who are experimenting with various tobacco and nicotine products. Not all of them will become regular users in adulthood and SES may be a key determinant of that transition^23^.

Another consideration in the evaluation of the SES differences we have found is the use of pocket money as a proxy for SES. Some studies have shown inconsistencies in the assumption that wealthier families have children with more disposable income; the opposite could be true in some settings^24^. The survey question we have used assessed disposable income; no data on family income and parental education or other SES indicators which could better reflect the overall individual and family circumstance were available. It is likely though that pocket money is more important than the broader family SES at this age. Students with more pocket money were more likely to report current use of cigarettes and electronic cigarettes in at least half of the countries, whereas only in one in five countries such differences were reported for smokeless tobacco. This might be directly linked to the actual cost and affordability of these products. Smokeless tobacco is subject to lower taxation compared to cigarettes globally and hence is more affordable^25^ for young people who usually depend on family to receive money. It is not a coincidence that the tobacco industry opposes tobacco control policies which prohibit the sale of individual cigarette sticks and manipulates prices to keep cheap cigarette brands in the market even when taxation increases^26^.

### Strengths and limitations

Our study benefits strongly from using surveys that employ nationally representative samples and consistent methodologies that enable between-country comparisons of tobacco and nicotine use. Although GYTS is mainly targeting adolescents aged 13–15 years, the age range and distribution in the samples varied between countries. Our use of age-adjusted models to compare prevalence in sub-groups reduces selection bias associated with differing age structures between countries and is more robust than reporting descriptive percentages of use.

However, our study is not without its limitations. GYTS only includes a limited number of sociodemographic variables and, hence, there may be residual confounding we could not account for. We were unable to include all countries worldwide and therefore provide an incomplete global assessment. Several larger countries, including China, Russia, and India, were omitted from this study as only sub-national surveys were available, whereas other high-income countries do not routinely conduct the GYTS. Our inclusion criteria included surveys as early as 2013, and the six-year range may prevent us from establishing a true cross-sectional picture of inequalities; however, we felt this was appropriately balanced against maximizing the number of included countries. Stratifying our findings by region resulted in small numbers (e.g. for the SEAR) so our regional comparisons should be interpreted with caution. Due to these limitations of our data, we decided against producing pooled regional and/or global estimates, which would not necessarily represent the differences by SES and sex globally. We also did not explore interactions between age, sex and SES due to the large number of countries assessed in this study; however, further country-level research may reveal more nuanced associations between these factors and nicotine use. Finally, we had no extensive data on products such as heated tobacco, nicotine pouches and flavored cigarettes which have recently gained substantial market shares in youth^27,^ ^28^.

## CONCLUSIONS

Our findings demonstrate a varied tobacco use inequalities landscape between countries and regions that have important policy implications. Tobacco control policies can be very effective, but they do not always have the same impact across population subgroups^11,29^. Smoking restrictions in schools and underage sales restrictions may be more effective in girls than boys, whereas boys may be more sensitive to price than girls^30^. Similarly, tobacco outlets are distributed unequally in society depending on income levels of communities^31^. In Europe, stronger tobacco control laws are consistently associated with lower smoking rates among high SES adolescents^32^. Taken together, this evidence base highlights the need for equity considerations to be at the forefront of tobacco control. Our analysis provides a useful context for local and international actors to design effective and equitable interventions and tobacco control policies to ensure population subgroups are not disproportionately affected by the harms of tobacco and nicotine use.

## Supplementary Material



## Data Availability

The data supporting this research are available from the following sources: https://nccd.cdc.gov/GTSSDataSurveyResources/Ancillary/Documentation.aspx?SUID=1&DOCT=1
