# Supplementary material for: Sociodemographic inequalities in cigarette, smokeless tobacco, waterpipe tobacco, and electronic cigarette use among adolescents aged 12–16 years in 114 countries: A cross-sectional analysis
Source: Tob Induc Dis. 2024 Sep 2;22:10.18332/tid/191824. doi: 10.18332/tid/191824 (PMC11367622; doi:10.18332/tid/191824)
Supplement: Supplementary file 1 [file TID-22-151-s1.pdf]

Figure S1. Prevalence ratios of current cigarette use in boys compared to girls among adolescents by country

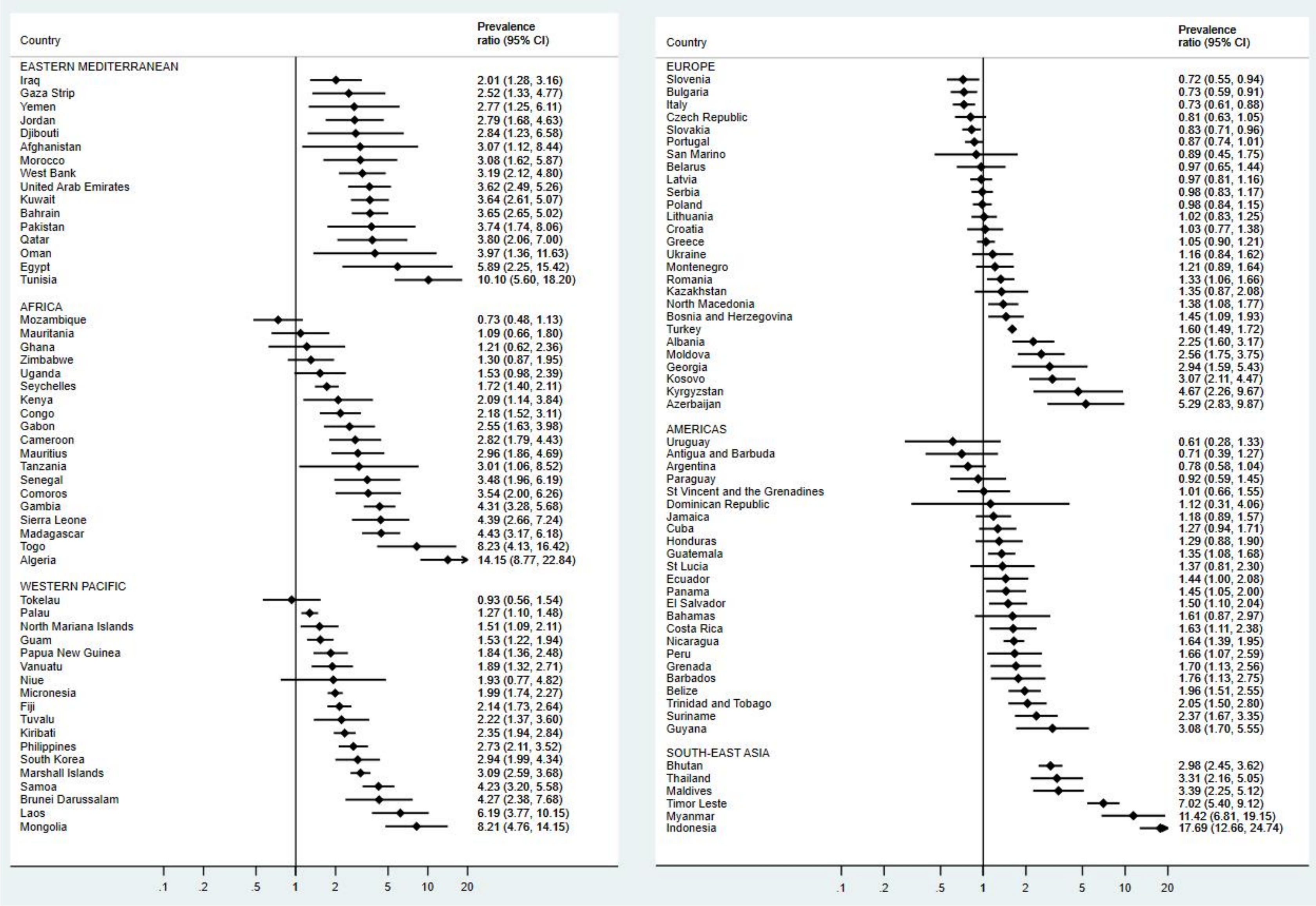

Prevalence ratios (PR) are from Poisson regression models adjusted for age and pocket money. PR>1 suggest higher prevalence among boys.

Figure S2. Prevalence ratios of current smokeless tobacco use in boys compared to girls among adolescents by country

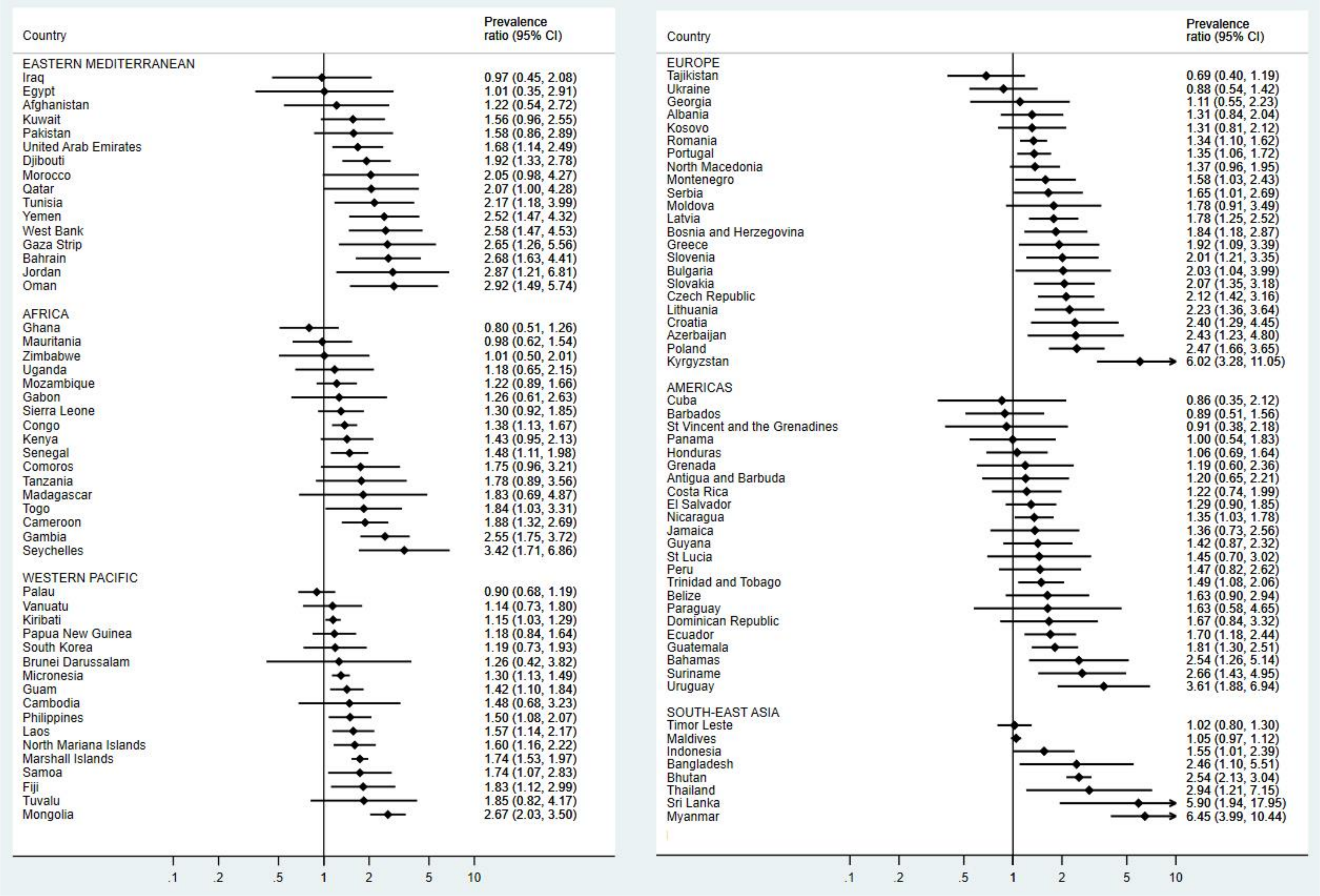

Prevalence ratios (PR) are from Poisson regression models adjusted for age and pocket money. PR>1 suggest higher prevalence among boys.

Figure S3. Prevalence ratios of current waterpipe tobacco use in boys compared to girls among adolescents by country

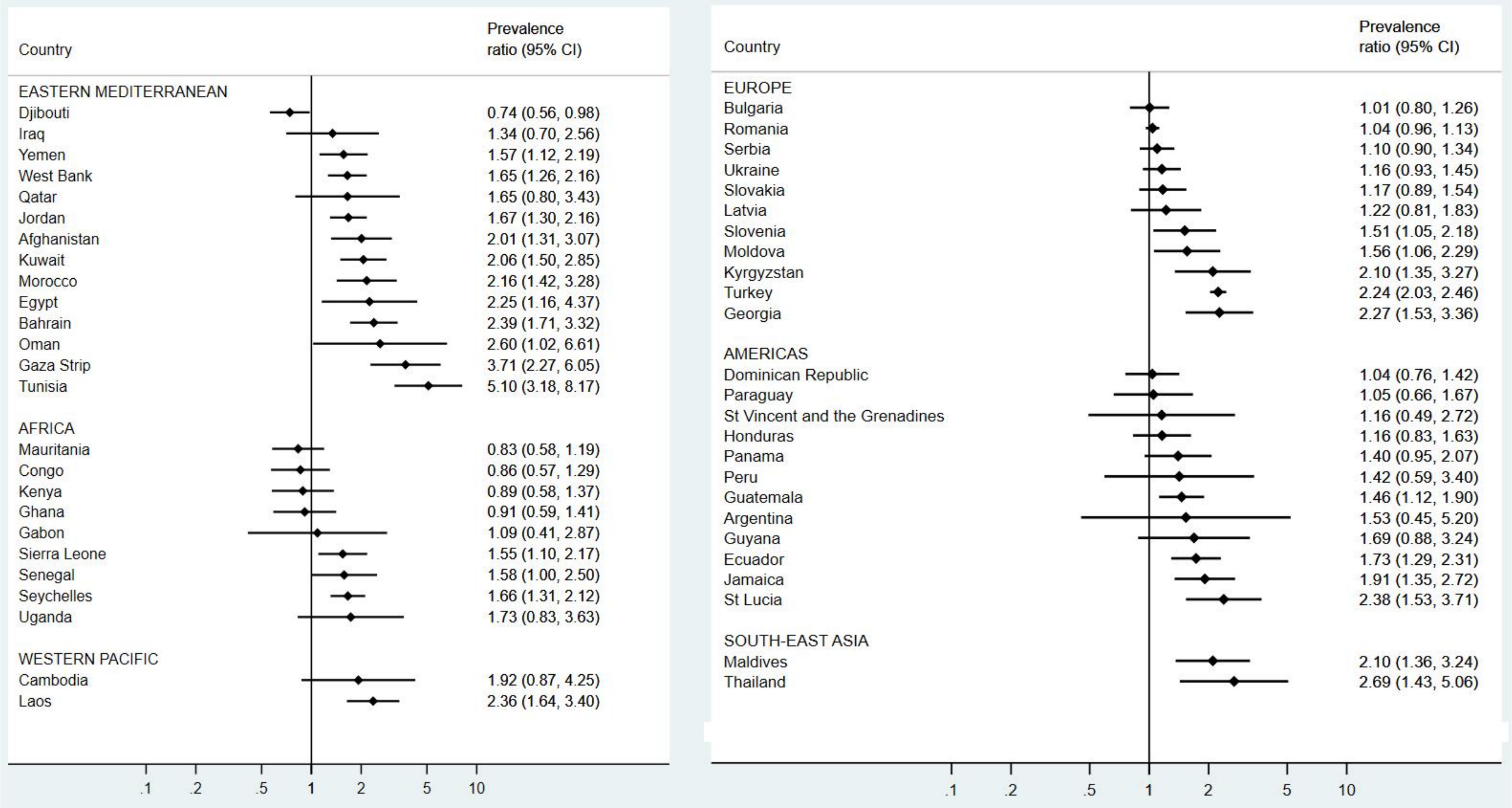

Prevalence ratios (PR) are from Poisson regression models adjusted for age and pocket money. PR>1 suggest higher prevalence among boys.

Figure S4. Prevalence ratios of current electronic cigarette use in boys compared to girls among adolescents by country

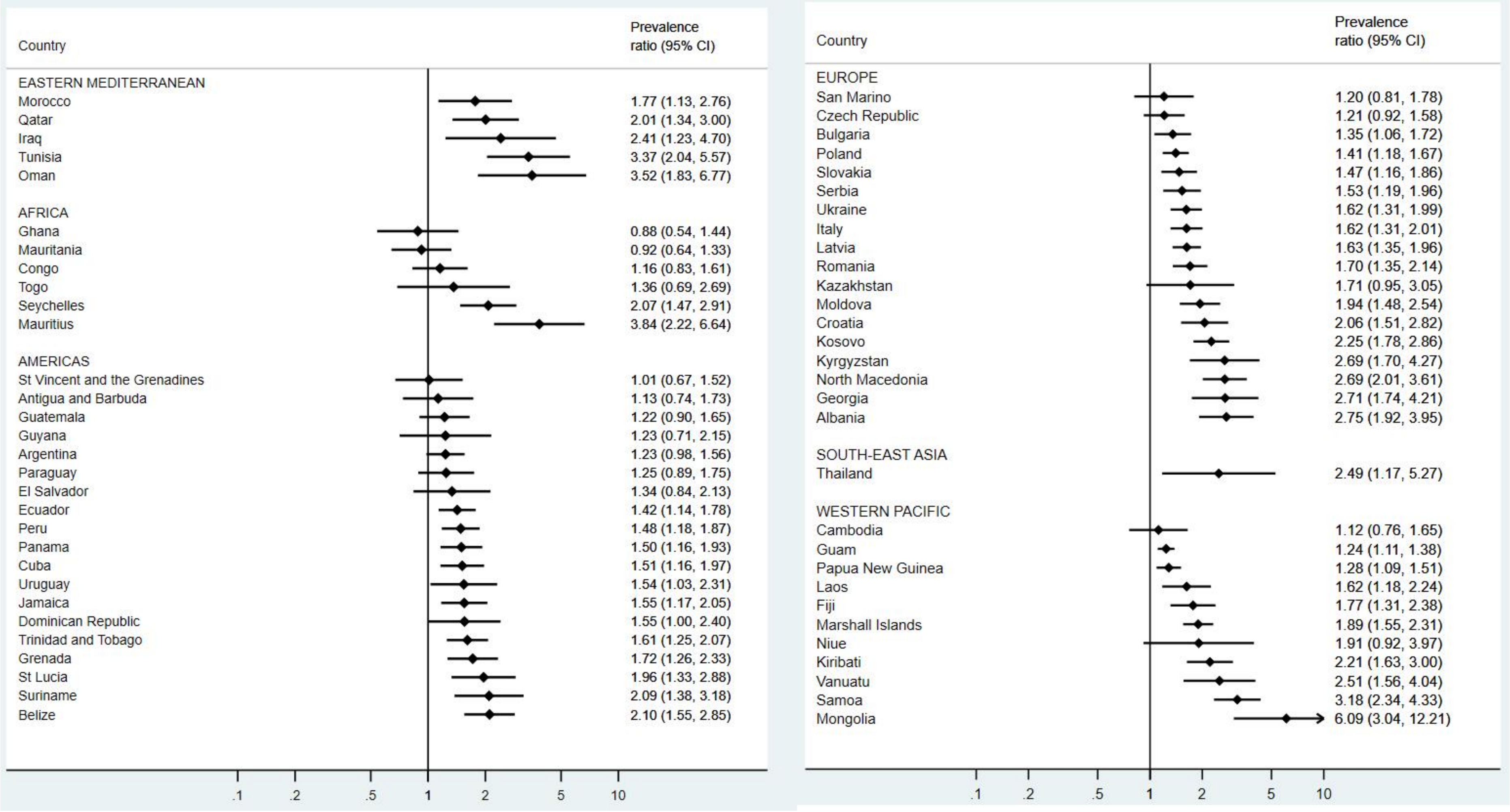

Prevalence ratios (PR) are from Poisson regression models adjusted for age and pocket money. PR>1 suggest higher prevalence among boys.

Figure S5. Prevalence ratios of current cigarette use in adolescents with high compared to low pocket money by country

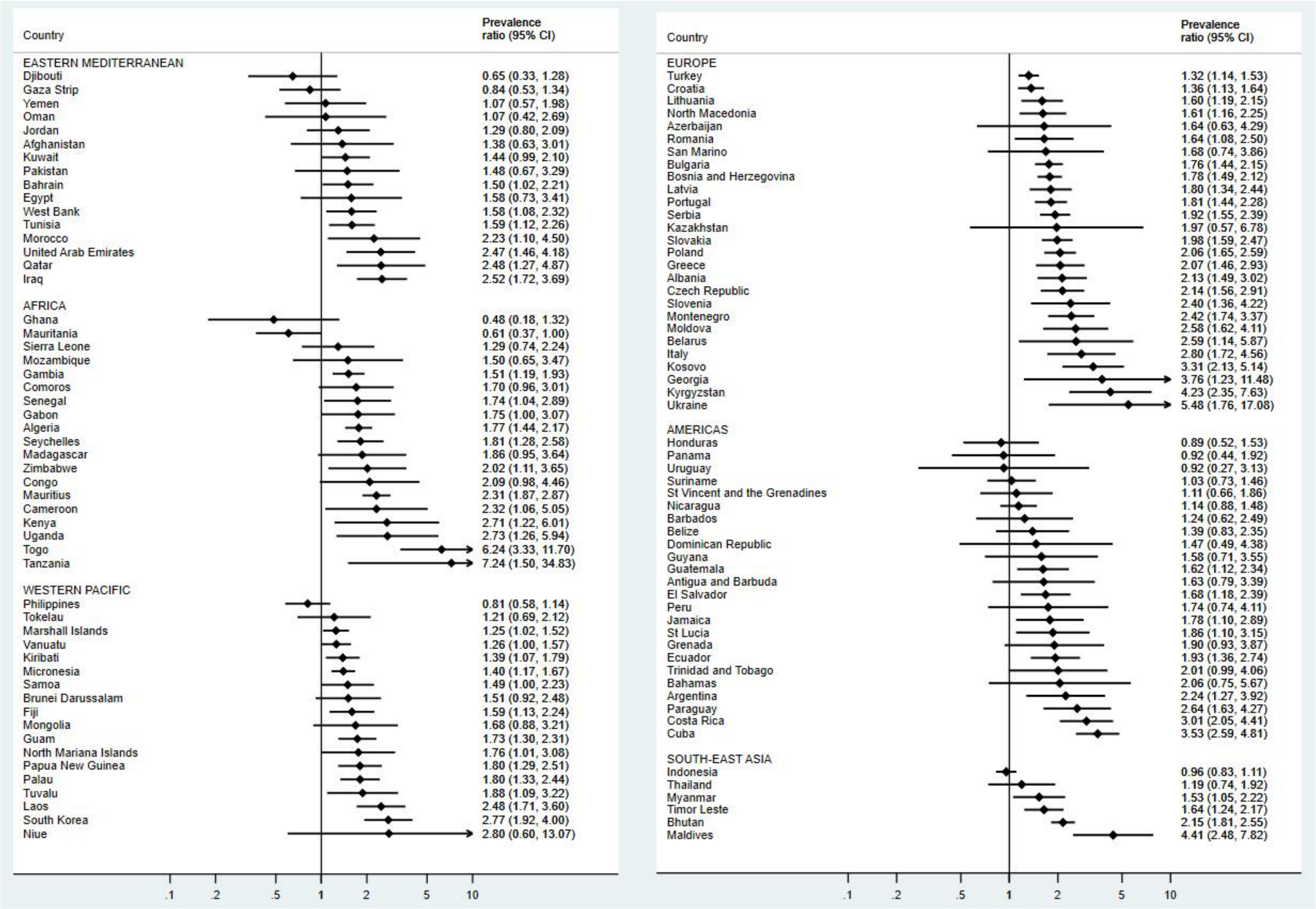

Prevalence ratios (PR) are from Poisson regression models adjusted for age and sex. PR>1 suggest higher prevalence among adolescent with high pocket money.

Figure S6. Prevalence ratios of current smokeless tobacco use in adolescents with high compared to low pocket money by country

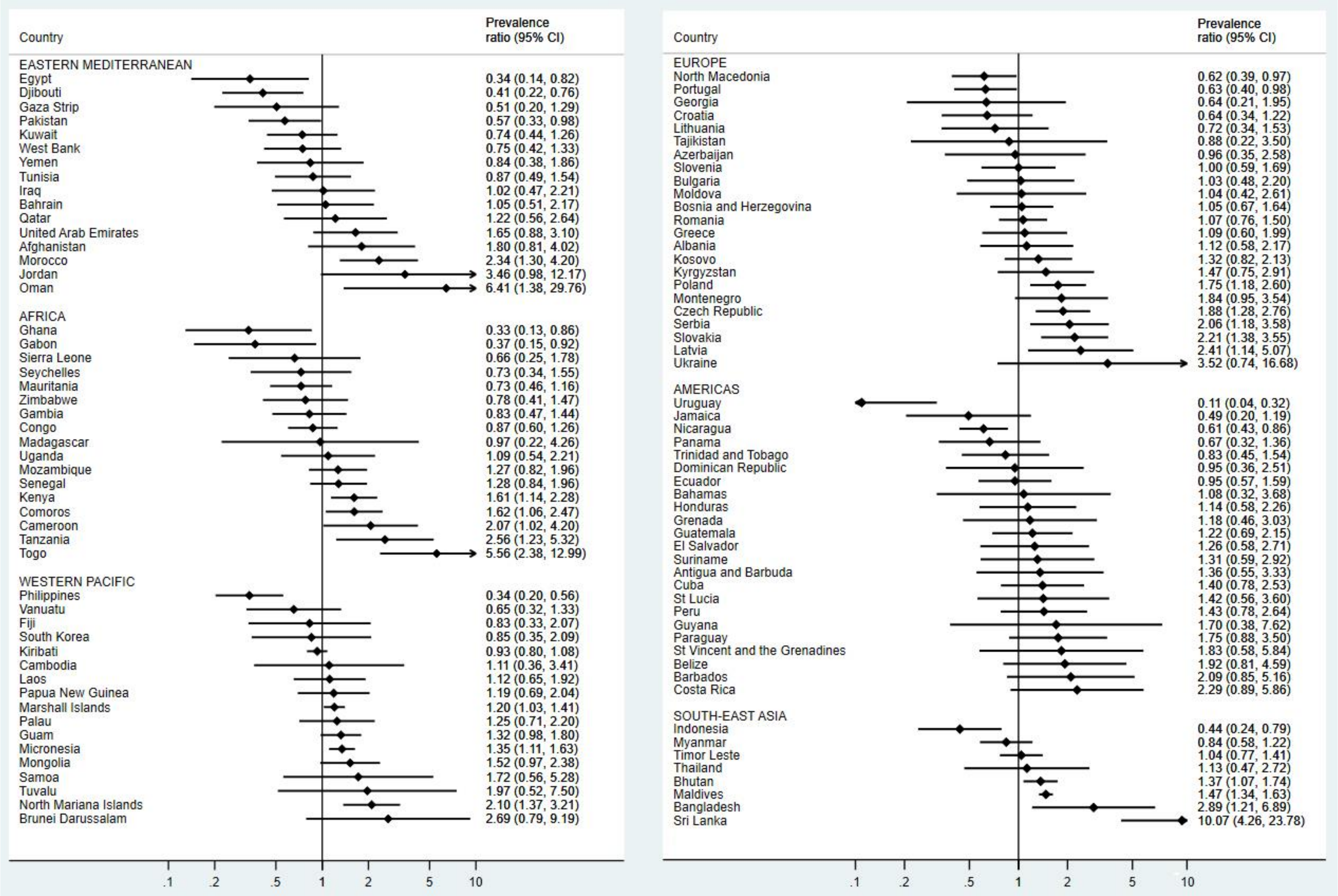

Prevalence ratios (PR) are from Poisson regression models adjusted for age and sex. PR>1 suggest higher prevalence among adolescent with high pocket money.

Figure S7. Prevalence ratios of current waterpipe tobacco use in adolescents with high compared to low pocket money by country

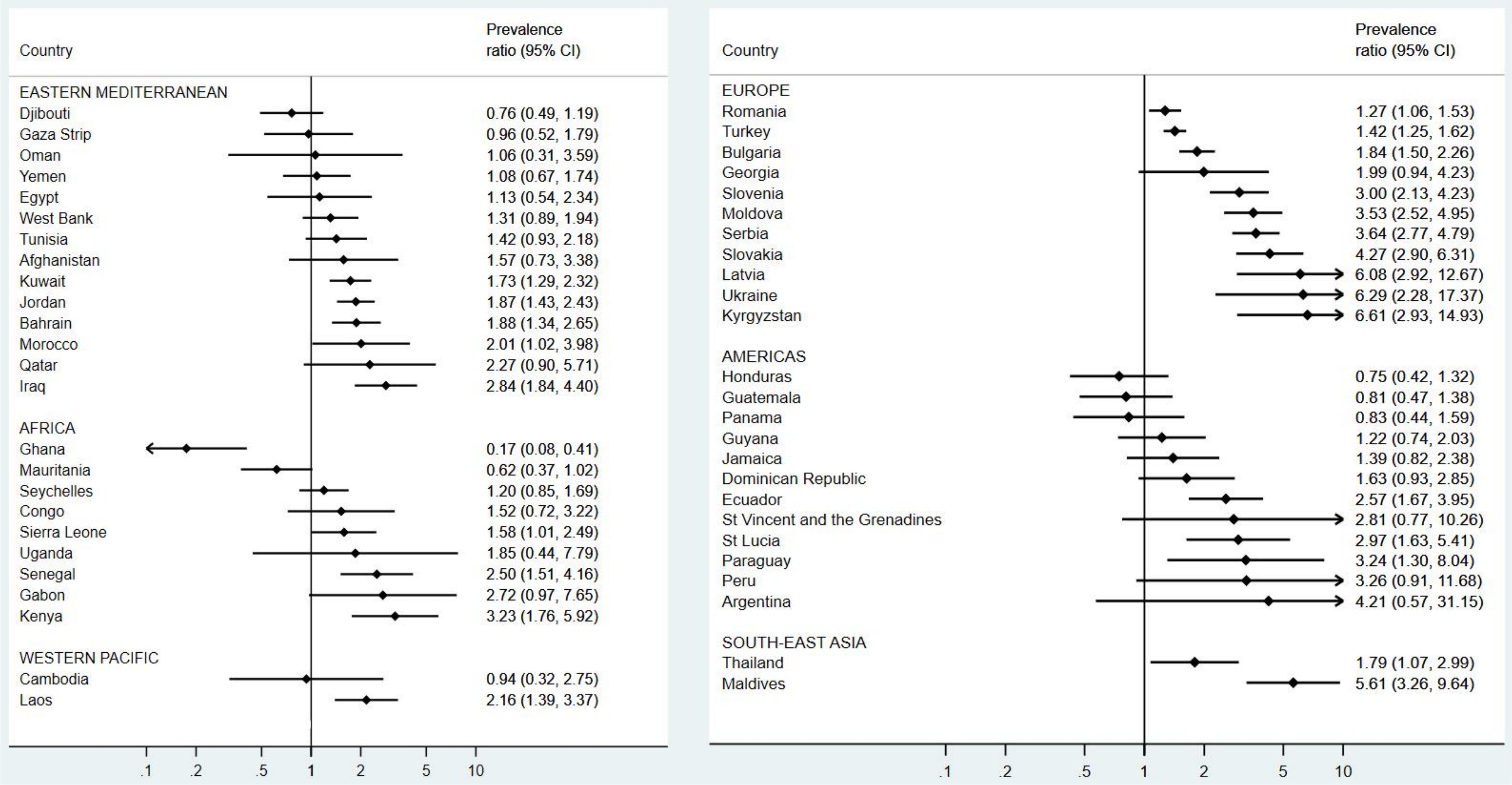

Prevalence ratios (PR) are from Poisson regression models adjusted for age and sex. PR>1 suggest higher prevalence among adolescent with high pocket money.

Figure S8. Prevalence ratios of current electronic cigarette use in adolescents with high compared to low pocket money by country

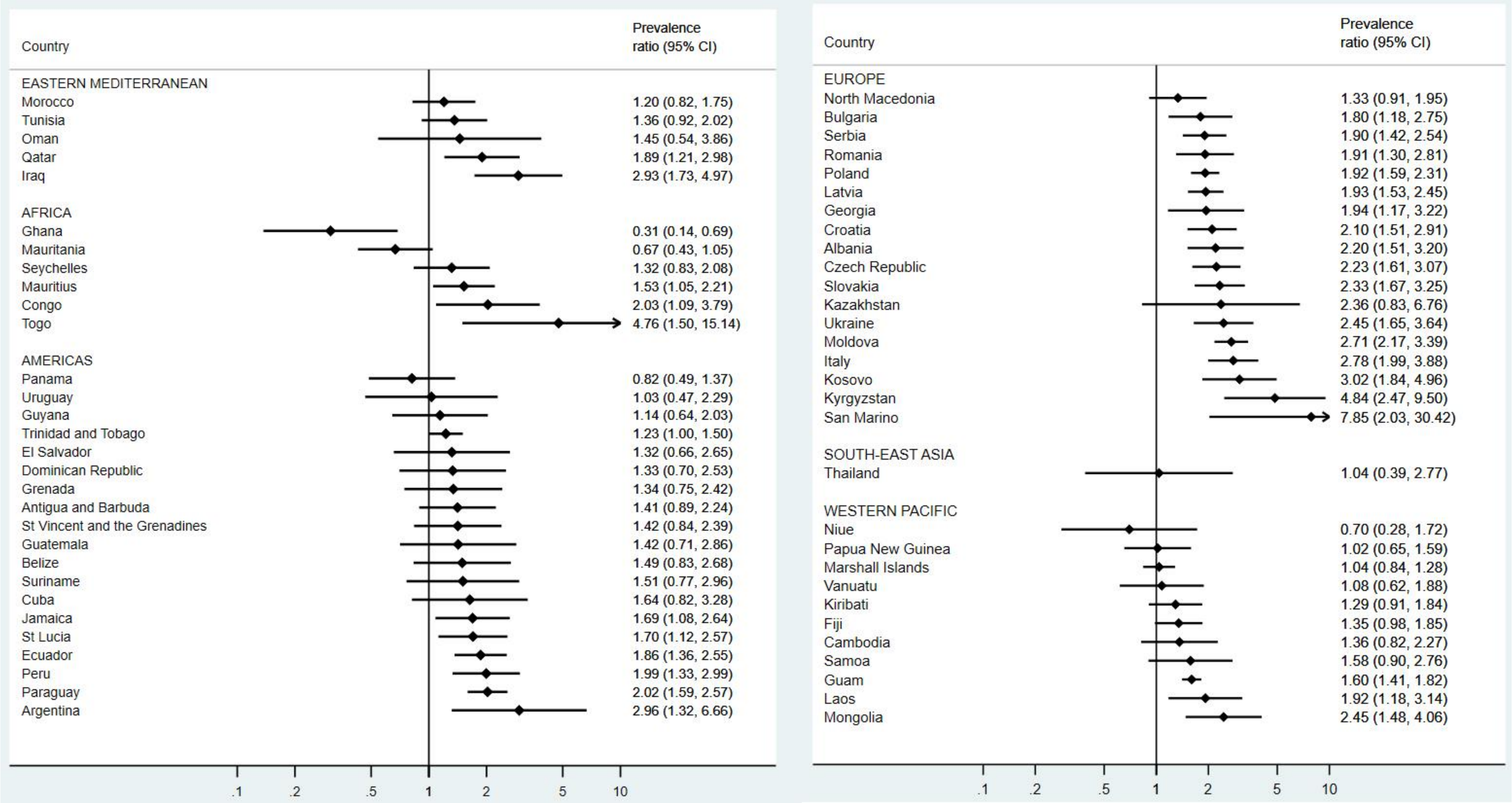

Prevalence ratios (PR) are from Poisson regression models adjusted for age and sex. PR>1 suggest higher prevalence among adolescent with high pocket money.

Table S1. Sample size and survey year for included countries (n=114)

Region	Country	Sample Size	Year	Region	Country	Sample Size	Year	Region	Country	Sample Size	Year
AFR	Algeria	6228	2013	AMR	St Lucia	1495	2017	EUR	North Macedonia	3474	2016
AFR	Cameroon	2922	2014	AMR	St Vincent & Grenadines	1519	2018	EUR	Poland	5154	2016
AFR	Comoros	2810	2015	AMR	Suriname	2212	2016	EUR	Portugal	11113	2013
AFR	Congo	6396	2019	AMR	Trinidad & Tobago	4128	2017	EUR	Romania	5409	2017
AFR	Gabon	1781	2014	AMR	Uruguay	2355	2019	EUR	San Marino	624	2018
AFR	Gambia	12585	2017	EMR	Afghanistan	1615	2017	EUR	Serbia	3861	2017
AFR	Ghana	5664	2017	EMR	Bahrain	3641	2015	EUR	Slovakia	3997	2016
AFR	Kenya	1895	2013	EMR	Djibouti	1818	2013	EUR	Slovenia	2629	2017
AFR	Madagascar	2920	2018	EMR	Egypt	2471	2014	EUR	Tajikistan	4083	2019
AFR	Mauritania	3740	2018	EMR	Gaza Strip	1536	2019	EUR	Turkey	122040	2017
AFR	Mauritius	4141	2016	EMR	Iraq	2560	2019	EUR	Ukraine	4065	2017
AFR	Mozambique	5599	2013	EMR	Jordan	2120	2014	SEAR	Bangladesh	3245	2013
AFR	Senegal	4320	2020	EMR	Kuwait	2477	2016	SEAR	Bhutan	4712	2019
AFR	Seychelles	2485	2015	EMR	Morocco	3915	2016	SEAR	Indonesia	9992	2019
AFR	Sierra Leone	6680	2017	EMR	Oman	2208	2016	SEAR	Maldives	4799	2019
AFR	Tanzania	3840	2016	EMR	Pakistan	8723	2013	SEAR	Myanmar	3633	2016
AFR	Togo	3917	2019	EMR	Qatar	2071	2018	SEAR	Sri Lanka	1505	2015
AFR	Uganda	3458	2018	EMR	Tunisia	2448	2017	SEAR	Thailand	1876	2015
AFR	Zimbabwe	6427	2014	EMR	United Arab Emirates	4259	2013	SEAR	Timor-Leste	5141	2019
AMR	Antigua & Barbuda	2268	2017	EMR	West Bank	1518	2016	WPR	Brunei Darussalam	1574	2013
AMR	Argentina	1453	2018	EMR	Yemen	2107	2014	WPR	Cambodia	3716	2016
AMR	Bahamas	1309	2013	EUR	Albania	4672	2015	WPR	Fiji	3697	2016
AMR	Barbados	1718	2013	EUR	Azerbaijan	2220	2016	WPR	Guam	2506	2017
AMR	Belize	1900	2014	EUR	Belarus	2992	2015	WPR	Kiribati	2622	2018
AMR	Costa Rica	3152	2013	EUR	Bosnia & Herzegovina	11536	2013	WPR	Laos	6550	2016
AMR	Cuba	4172	2018	EUR	Bulgaria	4042	2015	WPR	Micronesia	3911	2013
AMR	Dominican Republic	1532	2016	EUR	Croatia	3250	2016	WPR	Marshall Islands	3522	2016
AMR	Ecuador	5462	2016	EUR	Czech Republic	3926	2016	WPR	Mongolia	4146	2019
AMR	El Salvador	3215	2015	EUR	Georgia	1345	2017	WPR	Niue	163	2019
AMR	Grenada	2133	2016	EUR	Greece	4618	2013	WPR	North Mariana Islands	2174	2014
AMR	Guatemala	4301	2015	EUR	Italy	1680	2018	WPR	Palau	1484	2017
AMR	Guyana	1697	2015	EUR	Kazakhstan	2083	2014	WPR	Papua New Guinea	2301	2016
AMR	Honduras	3515	2016	EUR	Kyrgyzstan	6145	2019	WPR	Philippines	8789	2015
AMR	Jamaica	1685	2017	EUR	Kosovo	5031	2016	WPR	Tokelau	130	2014
AMR	Nicaragua	8735	2019	EUR	Latvia	4226	2019	WPR	Samoa	2076	2017
AMR	Panama	2621	2017	EUR	Lithuania	3030	2018	WPR	South Korea	4235	2013
AMR	Paraguay	4698	2019	EUR	Moldova	4717	2019	WPR	Tuvalu	764	2018
AMR	Peru	4148	2019	EUR	Montenegro	4216	2018	WPR	Vanuatu	2257	2017

AFR: African Region, AMR: Americas Region, SEAR: South-East Asian Region, EUR: European Region, EMR: Eastern Mediterranean Region, WPR: Western Pacific Region

Table S2. Poisson regression results on inequalities of cigarette, smokeless tobacco, waterpipe and e-cigarette use among adolescents by country.

Region	Country	Sex with higher prevalence of use				Socio-economic status (SES) with higher prevalence of use			
		**Cigarettes**	**Smokeless**	**Waterpipes**	**E-cigarettes**	**Cigarettes**	**Smokeless**	**Waterpipe**	**E-cigarettes**
AFR	Algeria	Boys				High SES			
AFR	Cameroon	Boys	Boys			High SES	High SES		
AFR	Comoros	Boys	No difference			No difference	High SES		
AFR	Congo	Boys	Boys	No difference	No difference	No difference	No difference	No difference	High SES
AFR	Gabon	Boys	No difference	No difference		High SES	Low SES	No difference	
AFR	Gambia	Boys	Boys			High SES	No difference		
AFR	Ghana	No difference	No difference	No difference	No difference	No difference	Low SES	Low SES	Low SES
AFR	Kenya	Boys	No difference	No difference		High SES	High SES	High SES	
AFR	Madagascar	Boys	No difference			No difference	No difference		
AFR	Mauritania	No difference	No difference	No difference	No difference	Low SES	No difference	No difference	No difference
AFR	Mauritius	Boys			Boys	High SES			No difference
AFR	Mozambique	No difference	No difference			No difference	No difference		
AFR	Senegal	Boys	Boys	No difference		High SES	No difference	High SES	
AFR	Seychelles	Boys	Boys	Boys	Boys	High SES	No difference	No difference	Not reported
AFR	Sierra Leone	Boys	No difference	Boys		No difference	No difference	High SES	
AFR	Tanzania	Boys	No difference			High SES	High SES		
AFR	Togo	Boys	Boys		No difference	High SES	High SES		High SES
AFR	Uganda	No difference	No difference	No difference		High SES	No difference	No difference	
AFR	Zimbabwe	No difference	No difference			High SES	No difference		
AMR	Antigua & Barbuda	No difference	No difference		No difference	No difference	No difference		No difference
AMR	Argentina	No difference		No difference	No difference	High SES		No difference	High SES
AMR	Bahamas	No difference	Boys			No difference	No difference		
AMR	Barbados	Boys	No difference			No difference	No difference		
AMR	Belize	Boys	No difference		Boys	No difference	No difference		No difference
AMR	Costa Rica	Boys	No difference			High SES	No difference		
AMR	Cuba	No difference	No difference		Boys	High SES	No difference		No difference

Region	Country	Sex with higher prevalence of use				Socio-economic status (SES) with higher prevalence of use			
		Cigarettes	Smokeless	Waterpipes	E-cigarettes	Cigarettes	Smokeless	Waterpipe	E-cigarettes
AMR	Dominican Republic	No difference	No difference	No difference	Boys	No difference	No difference	No difference	No difference
AMR	Ecuador	No difference	Boys	Boys	Boys	High SES	No difference	High SES	High SES
AMR	El Salvador	Boys	No difference		No difference	High SES	No difference		No difference
AMR	Grenada	Boys	No difference		Boys	No difference	No difference		No difference
AMR	Guatemala	Boys	Boys	Boys	No difference	High SES	No difference	No difference	No difference
AMR	Guyana	Boys	No difference	No difference	No difference	No difference	No difference	No difference	No difference
AMR	Honduras	No difference	No difference	No difference		No difference	No difference	No difference	
AMR	Jamaica	No difference	No difference	Boys	Boys	High SES	Low SES	No difference	High SES
AMR	Nicaragua	Boys	Boys			No difference	No difference		
AMR	Panama	Boys	No difference	No difference	Boys	No difference	No difference	No difference	No difference
AMR	Paraguay	No difference	No difference	No difference		High SES	No difference	High SES	High SES
AMR	Peru	Boys	No difference	No difference	Boys	No difference	No difference	No difference	High SES
AMR	St Lucia	No difference	No difference	Boys	Boys	High SES	No difference	High SES	High SES
AMR	St Vincent & the Grenadines	No difference	No difference	No difference		No difference	No difference	No difference	No difference
AMR	Suriname	Boys	Boys		Boys	No difference	No difference		No difference
AMR	Trinidad and Tobago	Boys	Boys		Boys	No difference	No difference		No difference
AMR	Uruguay	No difference	Boys		Boys	No difference	Low SES		No difference
EMR	Afghanistan	Boys	No difference	Boys		No difference	No difference	No difference	
EMR	Bahrain	Boys	Boys	Boys		High SES	No difference	High SES	
EMR	Djibouti	Boys	Boys	Girls		No difference	Low SES	No difference	
EMR	Egypt	Boys	No difference	Boys		No difference	Low SES	No difference	
EMR	Gaza Strip	Boys	Boys	Boys		No difference	No difference	No difference	
EMR	Iraq	Boys	No difference	No difference	Boys	High SES	No difference	High SES	High SES
EMR	Jordan	Boys	Boys	Boys		No difference	No difference	High SES	
EMR	Kuwait	Boys	No difference	Boys		No difference	No difference	High SES	
EMR	Morocco	Boys	No difference	Boys	Boys	High SES	High SES	High SES	No difference
EMR	Oman	Boys	Boys	Boys	Boys	No difference	High SES	No difference	No difference
EMR	Pakistan	Boys	No difference			No difference	Low SES		
EMR	Qatar	Boys	No difference	No difference	Boys	High SES	No difference	No difference	High SES

Region	Country	Sex with higher prevalence of use				Socio-economic status (SES) with higher prevalence of use			
		Cigarettes	Smokeless	Waterpipes	E-cigarettes	Cigarettes	Smokeless	Waterpipe	E-cigarettes
EMR	Tunisia	Boys	Boys	Boys	Boys	High SES	No difference	No difference	No difference
EMR	United Arab Emirates	Boys	Boys			High SES	No difference		
EMR	West Bank	Boys	Boys	Boys		High SES	No difference	No difference	
EMR	Yemen	Boys	Boys	Boys		No difference	No difference	No difference	
EUR	Albania	Boys	No difference		Boys	High SES	No difference		High SES
EUR	Azerbaijan	Boys	Boys			No difference	No difference		
EUR	Belarus	No difference				High SES			
EUR	Bosnia & Herzegovina	Boys	Boys			High SES	No difference		
EUR	Bulgaria	Girls	Boys	No difference	Boys	High SES	No difference	High SES	High SES
EUR	Croatia	No difference	Boys		Boys	High SES	No difference		High SES
EUR	Czech Republic	No difference	Boys		No difference	High SES	High SES		High SES
EUR	Georgia	Boys	No difference	Boys	Boys	High SES	No difference	No difference	High SES
EUR	Greece	No difference	Boys			High SES	No difference		
EUR	Italy	Girls			Boys	High SES			High SES
EUR	Kazakhstan	No difference			No difference	No difference			No difference
EUR	Kosovo	Boys	No difference		Boys	High SES	No difference		High SES
EUR	Kyrgyzstan	Boys	Boys	Boys	Boys	High SES	No difference	High SES	High SES
EUR	Latvia	No difference	Boys	No difference	Boys	High SES	High SES	High SES	High SES
EUR	Lithuania	No difference	Boys			High SES	No difference		
EUR	Moldova	Boys	No difference	Boys	Boys	High SES	No difference	High SES	High SES
EUR	Montenegro	No difference	Boys			High SES	No difference		
EUR	North Macedonia	Boys	No difference		Boys	High SES	Low SES		No difference
EUR	Poland	No difference	Boys		Boys	High SES	High SES		High SES
EUR	Portugal	No difference	Boys			High SES	Low SES		
EUR	Romania	Boys	Boys	No difference	Boys	High SES	No difference	High SES	High SES
EUR	San Marino	No difference			No difference	No difference			High SES
EUR	Serbia	No difference	Boys	No difference	Boys	High SES	High SES	High SES	High SES
EUR	Slovakia	Girls	Boys	No difference	Boys	High SES	High SES	High SES	High SES
EUR	Slovenia	Girls	Boys	Boys		High SES	No difference	High SES	

Region	Country	Sex with higher prevalence of use				Socio-economic status (SES) with higher prevalence of use			
		Cigarettes	Smokeless	Waterpipes	E-cigarettes	Cigarettes	Smokeless	Waterpipe	E-cigarettes
EUR	Tajikistan		No difference				No difference		
EUR	Turkey	Boys		Boys		High SES		High SES	
EUR	Ukraine	No difference	No difference	No difference	Boys	High SES	No difference	High SES	High SES
SEAR	Bangladesh		Boys				High SES		
SEAR	Bhutan	Boys	Boys			High SES	High SES		
SEAR	Indonesia	Boys	Boys			No difference	Low SES		
SEAR	Maldives	Boys	No difference	Boys		High SES	High SES	High SES	
SEAR	Myanmar	Boys	Boys			High SES	No difference		
SEAR	Sri Lanka		Boys				High SES		
SEAR	Thailand	Boys	Boys	Boys	Boys	No difference	No difference	High SES	No difference
SEAR	Timor-Leste	Boys	No difference			High SES	No difference		
WPR	Brunei Darussalam	Boys	No difference			No difference	No difference		
WPR	Cambodia		No difference	No difference	No difference		No difference	No difference	No difference
WPR	Fiji	Boys	Boys		Boys	High SES	No difference		No difference
WPR	Guam	Boys	Boys		Boys	High SES	No difference		High SES
WPR	Kiribati	Boys	Boys		Boys	High SES	No difference		No difference
WPR	Laos	Boys	Boys	Boys	Boys	High SES	No difference	High SES	High SES
WPR	Marshall Islands	Boys	Boys		Boys	High SES	High SES		No difference
WPR	Micronesia	Boys	Boys			High SES	High SES		
WPR	Mongolia	Boys	Boys		Boys	No difference	No difference		High SES
WPR	Niue	No difference			No difference	No difference			No difference
WPR	North Mariana Islands	Boys	Boys			High SES	High SES		
WPR	Palau	Boys	No difference			High SES	No difference		
WPR	Papua New Guinea	Boys	No difference		Boys	High SES	No difference		No difference
WPR	Philippines	Boys	Boys			No difference	Low SES		
WPR	Samoa	Boys	Boys		Boys	High SES	No difference		No difference
WPR	South Korea	Boys	No difference			High SES	No difference		
WPR	Tokelau	No difference				No difference			
WPR	Tuvalu	Boys	No difference			High SES	No difference		

Region	Country	Sex with higher prevalence of use				Socio-economic status (SES) with higher prevalence of use			
		Cigarettes	Smokeless	Waterpipes	E-cigarettes	Cigarettes	Smokeless	Waterpipe	E-cigarettes
WPR	Vanuatu	Boys	No difference		Boys	High SES	No difference		No difference

Footnote: Results shown for inequalities by sex are from Poisson regression models adjusted for age and socio-economic status (SES). Results shown for inequalities by SES are from Poisson regression models adjusted for age and sex.

© 2024 Jawad M. et al.
